# Microplastic biofilms as potential hotspots for plastic biodegradation and nitrogen cycling: a metagenomic perspective

**DOI:** 10.1093/femsec/fiaf035

**Published:** 2025-04-02

**Authors:** Samantha G Fortin, Kelley Uhlig, Robert C Hale, Bongkeun Song

**Affiliations:** Virginia Institute of Marine Science, William and Mary, Gloucester Point, Virginia 23062, United States; Virginia Institute of Marine Science, William and Mary, Gloucester Point, Virginia 23062, United States; Virginia Institute of Marine Science, William and Mary, Gloucester Point, Virginia 23062, United States; Virginia Institute of Marine Science, William and Mary, Gloucester Point, Virginia 23062, United States

**Keywords:** biofilm, biopolymer, microplastics, nitrogen cycling, plastic degradation, plastisphere

## Abstract

Microplastics are an emerging contaminant worldwide, with the potential to impact organisms and facilitate the sorption and release of chemicals. Additionally, they create a novel habitat for microbial communities, forming biofilms known as the plastisphere. While the plastisphere has been studied in select aquatic environments, those in estuarine ecosystems merit additional attention due to their proximity to plastic debris sources. Additionally, the role plastisphere communities play in nutrient cycling has rarely been examined. This study used metagenomic analysis to investigate the taxonomic composition and functional genes of developing plastisphere communities living on petroleum-based (polyethylene and polyvinyl chloride) and biopolymer-based (polylactic acid) substrates. Isolated metagenome-assembled genomes (MAGs) showed plastisphere communities have the genes necessary to perform nitrification and denitrification and degrade petroleum and biopolymer-based plastics. The functions of these plastispheres have implications for estuarine nitrogen cycling and provide a possible explanation for the plastisphere microbes’ competitiveness in biofilm environments. Overall, microplastics in the estuarine system provide a novel habitat for microbial communities and associated nitrogen cycling, facilitating the growth of microbes with plastic-degrading capabilities.

## Teaser

Biofilm communities growing on microplastic particles made of petroleum and biopolymers include nitrogen-cycling and plastic-degrading microbes.

## Introduction

From 2000 to 2019, global plastic waste doubled (OECD 2022) and the environmental accumulation rate continues to escalate. Contamination of marine and freshwater ecosystems by plastic debris has followed suit. Plastic debris is now found from the deep sea (Van Cauwenberghe et al. 2013) to coastal estuaries (Yonkos et al. 2014). The impact of microplastics, particles between 1 µm and 5 mm in diameter (Arthur et al. 2009, Cole and Galloway 2015), on ecosystems has been the focus of an increasing number of studies; however, many questions and aspects of the interaction between microplastics and the environment still need to be addressed.

Microplastics are known to impact a wide range of marine organisms from oyster larvae (Cole and Galloway 2015) and zooplankton (Cole et al. 2013) to adult bivalves, crustaceans, fish (Rochman et al. 2015, Setälä et al. 2016), and marine mammals (Merrill et al. 2023), leading to starvation, damage to internal organs, altered feeding behavior and energy allocation, enhanced disease susceptibility, and decreased reproductive success (Galloway and Lewis 2016, Sussarellu et al. 2016, Seeley et al. 2023). Additional concerns regarding microplastics include the sorption of persistent organic pollutants and the leaching of toxic chemical additives like flame retardants and plasticizers (Hartmann et al. 2017, Hermabessiere et al. 2017, Paluselli and Kim 2020). Furthermore, plastic particles provide a substrate for biofilm formation, allowing microplastics to carry and transport pathogens and invasive species (Zettler et al. 2013, Kirstein et al. 2016).

The biofilm community on microplastics has been dubbed the plastisphere (Zettler et al. 2013). It has been examined using 16S rRNA gene sequencing of microplastics collected from the open ocean, coastal ecosystems, and rivers, though the majority of plastisphere studies have been performed in pelagic marine environments (Zettler et al. 2013, McCormick et al. 2014, Amaral-Zettler et al. 2015, Oberbeckmann et al. 2015, Zhai et al. 2023). Biofilms begin to form on plastic particles in marine systems rapidly with visible biofilms forming in less than a week (Lobelle and Cunliffe 2011). After about a month, plastic biofilm communities reach a stable bacterial abundance and can be considered mature, as opposed to being in the colonization or growth phases (Cheng et al. 2021). These communities are distinct from microbial communities in the surrounding seawater and those on substrates such as glass, wood, and stone (Zettler et al. 2013, Oberbeckmann et al. 2015, McCormick et al. 2016, Miao et al. 2018, Cheng et al. 2021).

Mature plastisphere communities are generally dominated by a mix of microbial eukaryotes, including diatoms and bryophytes, and prokaryotes, such as *Alphaproteobacteria, Gammaproteobacteria, Bacteriodetes*, and *Cyanobacteria* (Oberbeckmann et al. 2014, Kirstein et al. 2019, Zhang et al. 2021, Zhai et al. 2023). Plastisphere communities have been found to vary with geographic location, season, and environmental variables like temperature and the presence of algal blooms. Plastic polymer type may also be influential, though its impact on the plastisphere community is still being debated and conflicting results have been reported (Zettler et al. 2013, Oberbeckmann et al. 2014, Amaral-Zettler et al. 2015, Oberbeckmann et al. 2015, Dussud et al. 2018, Kirstein et al. 2019, Cheng et al. 2021, Zhang et al. 2021). Most plastisphere research to date has focused on polyethylene (PE), polypropylene (PP), or polystyrene (PS) (Zhai et al. 2023). Other types, including the common polyvinyl chloride (PVC) and increasingly prevalent and promoted biopolymers [e.g. polylactic acid (PLA)], have been less studied (Zhai et al. 2023). In the few investigations that have compared petroleum-based polymers and biopolymers, slight differences have been observed in mature community composition (Dussud et al. 2018, Kirstein et al. 2019, Cheng et al. 2021).

The plastisphere has become an increasing focus of research due to concerns regarding pathogens and the spread of invasive species (Zettler et al. 2013, Keswani et al. 2016, Kirstein et al. 2016) as well as a growing interest in identifying plastic-degrading organisms (Zettler et al. 2013, Bryant et al. 2016, Zhai et al. 2023). However, most studies have focused on the taxonomic composition of the plastisphere. Only a few studies have examined community metabolism and functional genes. Bryant et al. (2016) examined the potential function of the organisms in water-column plastisphere communities. PE and PP particles from the Pacific Ocean were found to have a high abundance of genes involved in nitrogen fixation, hydrocarbon degradation, surface adhesion, secretion, iron transportation, and phosphonate utilization (Bryant et al. 2016). Further examination of the same samples showed enrichment of metal and antibiotic resistance genes in the plastisphere (Yang et al. 2019).

The few studies examining the potential role of microplastics in nutrient metabolism have focused on nitrogen cycling in sediments (Seeley et al. 2020, Wang et al. 2024). In sediment microcosm experiments, the addition of PE, PLA, and polyurethane foam (PUF) increased the abundance of genes encoding for enzymes involved in nitrification and denitrification and elevated the rate of sediment denitrification (Seeley et al. 2020). However, the addition of PVC microplastics decreased rates of denitrification and associated gene abundances (Seeley et al. 2020). Another study in sediment microcosms with PS and PE microplastics found no effect on denitrification genes, an increase in nitrification genes, and a decrease in genes for dissimilatory nitrate reduction to ammonium (DNRA) (Wang et al. 2024). Despite the varied effect of microplastics on sediment nitrogen cycling, microplastics may have a significant impact on nitrogen cycling in natural environments.

Although the plastisphere is known to harbor a unique community structure, exhibit distinct metabolic capabilities, and potentially influence nitrogen cycling, few studies have employed metagenomic techniques to investigate the functional capabilities of microbes living in plastisphere communities on biopolymers or the effects of early colonization when considering the metabolic potential of the plastisphere community. This study aims to address this by applying metagenomic analysis to identify both the microbial taxa and the functional genes of communities forming on PLA, PE, and PVC in a coastal estuary. We report the potential role of the plastisphere in estuarine nitrogen cycling and the genetic evidence of plastic-degradation potential in the plastisphere community. Additionally, we examine the changes in taxonomic structure and functional genes throughout the first month of plastisphere growth, allowing an examination of changes during biofilm maturation.

## Materials and methods

### Deployment and experimental setup

Three polymers, including the petroleum-based polymers high-density polyethylene (PE; Rigidex HD6070EA, Goodfellow Cambridge Ltd.) and polyvinyl chloride (PVC; Geon Vinyl Rigid Extrusion 6935, PolyOne Corp.) and the biopolymer polylactic acid (PLA; 100% Natural Virgin IC3D, LLC), were selected. PE is the most commonly found microplastic in Chesapeake Bay (Yonkos et al. 2014, Bikker et al. 2020), and PE microplastics have been found in the effluent of the York River Wastewater Treatment Plant (Fortin et al. 2019). PVC, while not commonly found as a microplastic in Chesapeake Bay (Bikker et al. 2020), is in the top three most common plastic polymers (PlasticsEurope 2017, 2024) and is frequently used in estuarine systems for in-water construction of docks and fishing equipment, resulting in large exposure of estuarine waters to PVC. PLA is tied with starch-based polymers for the most common plastic biopolymer, and its production is increasing (Fredi and Dorigato 2021, PlasticsEurope 2024), potentially allowing it to be a future microplastic contaminant to estuarine systems. All three polymers are often used to produce the single-use items that are the most commonly found macroplastics in Chesapeake Bay (PlasticsEurope 2017, Hale et al. 2020, Fredi and Dorigato 2021).

Two grams of each polymer, in the form of 2–4 mm beads, were placed in nine individual fiberglass mesh bags (27 total for all polymers) and deployed in the water column of the York River estuary, Virginia, USA (37°14′49.1“ N, 76°30′03.5″ W) beginning on July 11, 2017. The bags were attached to a buoy line and submerged within the photic zone according to the specific gravity of each plastic (i.e. PE was closest to the surface, followed by PLA, then PVC). Triplicate bags (A, B, and C) of each polymer were collected after 7, 14, and 28 days. Once retrieved, the bags were rinsed with deionized water to remove debris and the microplastic beads were removed, transferred to 15 ml Falcon tubes, and stored at −20°C.

Water quality data was collected from the nearby (37°14′50.2′' N, 76° 29′ 57.7′' W) Gloucester Point continuous monitoring station (Station YRK005.40) maintained by the Virginia Estuarine and Coastal Observing System (CBNEER-VA VIMS 2017).

### Metagenomic sequencing and bioinformatics

DNA extraction was performed on the beads (PE: 20 beads, PVC: 15 beads, PLA: 10 beads) in triplicate for each polymer type and timepoint using the DNEasy PowerSoil Kit (Qiagen) following the manufacturer’s protocols. Due to their relatively large size, the bead-beating step was modified, and the microplastic beads were placed in 15 ml Falcon tubes with the contents of two PowerSoil garnet bead-beating tubes and vortexed for 10 min. Extracted DNA was then shipped to Novogene Co, Ltd. for metagenomic sequencing on the Illumina platform. The quality of metagenomic sequences was checked with FastQC version 0.11.5 (Andrews 2010) and poor-quality reads were trimmed or removed with Trimmomatic version 0.36 (Bolger et al. 2014).

Metagenomic sequences were assigned to taxonomic ranks based on results from the Kaiju webserver (Menzel et al. 2016) and the NCBI Blast nr +euk database. Taxonomic classifications and community structure were then analyzed, using the phyloseq package (McMurdie and Holmes 2013) and R version 4.0.3 (R Core Team 2018), with a principal component analysis using a Bray–Curtis distance matrix. Rarefaction curves were calculated using the vegan package (Oksanen et al. 2018). All figures were made using ggplot2 (Wickham 2005).

Reads were uploaded to the KBase platform (Arkin et al. 2018) and replicate samples A, B, and C were combined to create one read library for each polymer and timepoint (Supplementary Table S1). Fama version 1.1.1 (Kazakov and Novichkov 2019) was used to identify nitrogen-cycling genes (Supplementary Table S2) and calculate the relative abundance of those genes in each sample. Fama calculates relative abundance using the number of fragments per kb of effective gene length per genome equivalent (efpkg) (Kazakov and Novichkov 2019). Fama combines *amoA* and *pmoA* as well as *narG* and *nxrA* since they are homologous genes. The efpkg for each sample was corrected to reflect only *amoA* or *narG* based on the taxonomy assigned to each identified *amoA/pmoA* or *narG/nxrA* gene. For example, if an individual *amoA/pmoA* gene was identified as belonging to a methanotrophic bacteria rather than an ammonia oxidizer, the efpkg for that specific gene was subtracted from the total *amoA* efpkg for the sample. *nxrA* was not included in the overall analysis because it was present in only one sample and had an extremely low efpkg, indicating that nitrite oxidation was not important in plastisphere communities.

The combined read libraries were then used to create one contig assembly for each polymer and timepoint (Supplementary Table S1) with MEGAHIT version 1.2.9 (Li et al. 2015). Assembled contigs were binned using three different binning programs: MaxBin2 version 2.2.4 (Wu et al. 2014), MetaBAT2 version 1.7 (Kang et al. 2019), and CONCOCT version 1.1 (Alneberg et al. 2014). The three sets of bins were then analyzed using DAS Tool version 1.1.2 (Sieber et al. 2018), and a final set of bins, or metagenome-assembled genomes (MAGs), was selected. The completeness and contamination of those MAGs were measured using CheckM version 1.0.18 (Parks et al. 2015). Only high-quality MAGs, i.e. those that were >70% complete and had <5% contamination (Parks et al. 2015), were included for further analysis. Fama was again used to annotate nitrogen-cycling genes present in the MAGs and the taxa represented by each MAG were identified with GTDB-Tk Classify v1.6.0 (Chaumeil et al. 2019). MAGs were determined to be MAGs related to nitrogen metabolism when, based on the Fama results, they contained at least one target gene for a nitrogen cycling process (Supplementary Table S2). Plastic-degrading MAGs were identified based on the presence of genes linked to plastic degradation. Plastic-degrading genes were identified using the PlasticDB database (Gambarini et al. 2022) accessed on 13 June 2023. The PlasticDB gene database was searched against all high-quality MAGs using BLAST+ (Camacho et al. 2009); gene hits were only accepted if >50% of the gene was aligned with >50% identity; all e-values were <0.001.

## Results

### Environmental conditions during the deployment

During the microplastic deployment, the York River stayed at a fairly consistent temperature and salinity, ranging between 25.4 and 31.1°C and 15.7 and 23.1 psu, respectively (Supplementary Table S3). There was an increase in chlorophyll *a* measurements (average of 15.3 compared to 6.7 and 7.6 in the first two weeks) in the last two weeks of the deployment (day 15–28). This was accompanied by an increase in turbidity, fluorescence, and dissolved oxygen (Supplementary Table S3).

### Plastisphere community composition

**Figure 1.**
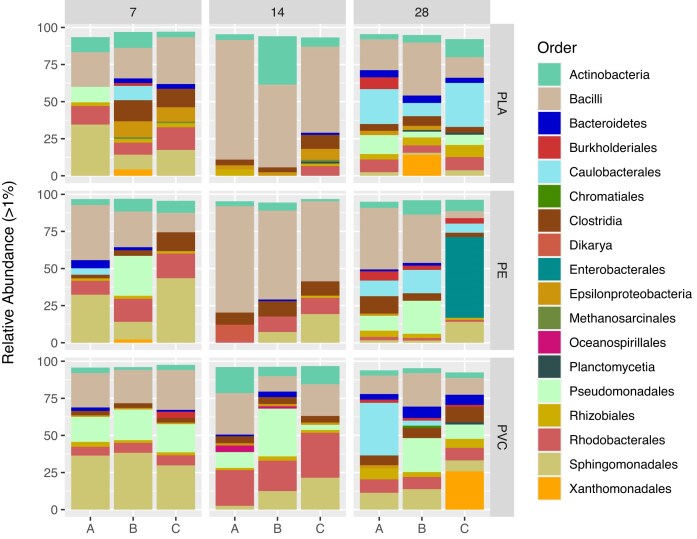
Relative abundance of orders >1% in each triplicate (A, B, and C) at each timepoint (7, 14, and 28 days) of the plastisphere communities present on PLA, PE, and PVC.

**Figure 2.**
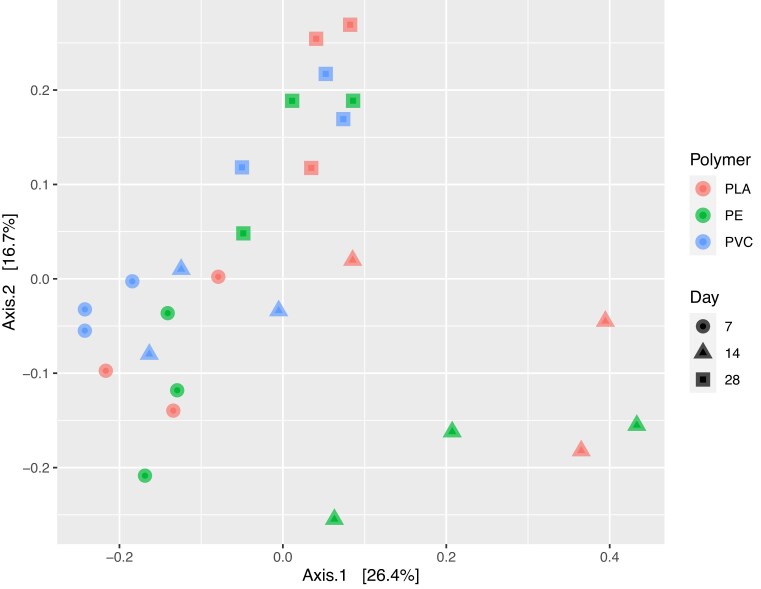
Principal component analysis representing beta diversity of the plastisphere community at each timepoint (7, 14, and 28 days) on PLA, PE, and PVC.

Plastisphere communities grew quickly on PE, PVC, and PLA microplastics in the York River. A visible biofilm layer was present on all plastic polymers by the day 7 sampling (data not shown). Day 7 plastisphere communities were dominated by *Bacilli* and *Sphingomonadales* (Fig. 1) and clustered closely together regardless of polymer type when considering beta diversity (Fig. 2). By day 14, the plastisphere communities were showing some divergence based on polymer type with PE and PLA separating from PVC, which remained clustered with the day 7 samples (Fig. 2). However, PERMANOVA statistical tests were unable to be performed due to a lack of homogeneously dispersed data. PE and PLA were heavily dominated by *Bacilli* on day 14, while PVC was dominated by *Rhodobacterales* and, in replicate B, *Pseudomonadales*, with a smaller relative abundance of *Bacilli* (Fig. 1). More mature biofilm communities were observed on day 28 samples. These were slightly separated from the communities present on previous days, though all polymer types were clustered together (Fig. 2). Based on the beta diversity, the PVC community changed the least from day 7 to day 28, while PE and PLA communities exhibited a greater change over time (Fig. 2). The diversity and complexity of the plastisphere communities generally increased over time and were similar across the three different polymers (Supplementary Fig. S1).

### Nitrogen-cycling genes in plastisphere metagenomes

**Figure 3.**
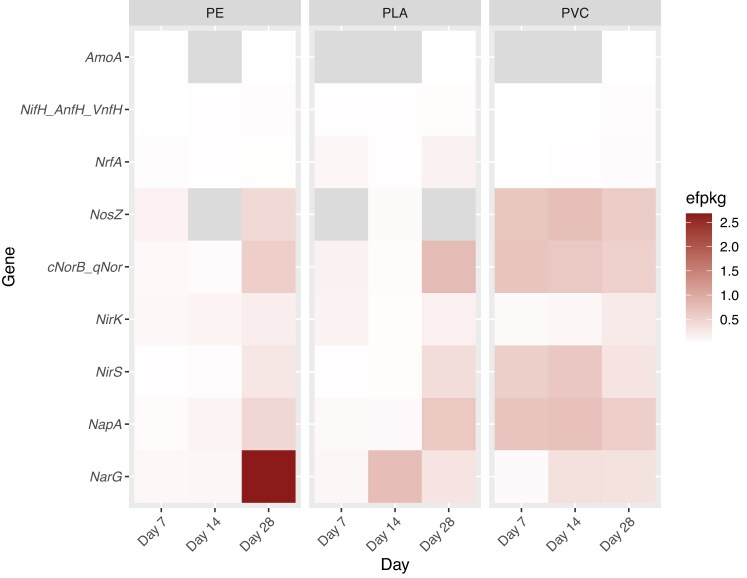
Heatmap showing the relative abundance, represented in efpkg, of nitrogen cycling genes in the metagenomes of plastispheres in each week of the experiment. Plastic polymers are PE, PLA, and PVC. The metagenomes for each week include the combined reads from three replicate plastisphere samples. Gray boxes show the absence of the gene in the plastisphere.

The potential role of plastisphere communities in the estuarine nitrogen cycle was investigated using common marker genes for nitrogen-cycling pathways (Supplementary Table S2). Genes that code for enzymes in the denitrification pathway were the most abundant nitrogen-cycling genes in all plastisphere communities (Fig. 3). The most abundant gene was *narG*, especially in the day 28 PE plastisphere. *narG* abundance generally increased over time in all plastisphere communities. *napA* was present in all plastisphere communities. Its abundance increased over time in PE and PLA communities, but in PVC communities, *napA* abundance was high in all communities and was higher than *narG* abundance (Fig. 3). Nitrite reductase genes *nirK* and *nirS*, as well as nitric-oxide reductase (*nor*), increased in abundance over time in PE and PLA communities. In PVC communities, *nirS* and *nor* remained high at all timepoints while *nirK* increased over time (Fig. 3). Overall, *nirS* was more abundant than *nirK*. Nitrous oxide reductase, *nosZ*, was present in PE communities only on days 7 and 28. Higher abundance of *nosZ* was observed on day 28. *nosZ* was present in PLA in very low relative abundance in only the day 14 plastisphere samples. In PVC communities, *nosZ* was present at all timepoints at a higher relative abundance than the other polymers (Fig. 3).

Genes for other nitrogen-cycling pathways were present in the plastisphere communities, though to a lesser degree. DNRA genes (*nrfA*) were found in all plastisphere communities at a low relative abundance. However, PLA plastispheres on day 7 and 28 had an elevated abundance of *nrfA* (Fig. 3). The marker gene for ammonia oxidation (*amoA*) was found in PE plastispheres only on day 7 and 14 and was found in PLA and PVC plastispheres only on day 28. Relative abundances of *amoA* were all very low (Fig. 3). Nitrogen fixation, using *nifH* as a marker gene, was found in all plastispheres, though at very low relative abundances (Fig. 3). Genes for anaerobic ammonium oxidation (anammox) were not found in the metagenome of any plastisphere community.

### MAGs associated with nitrogen cycling or plastic degradation

**Figure 4.**
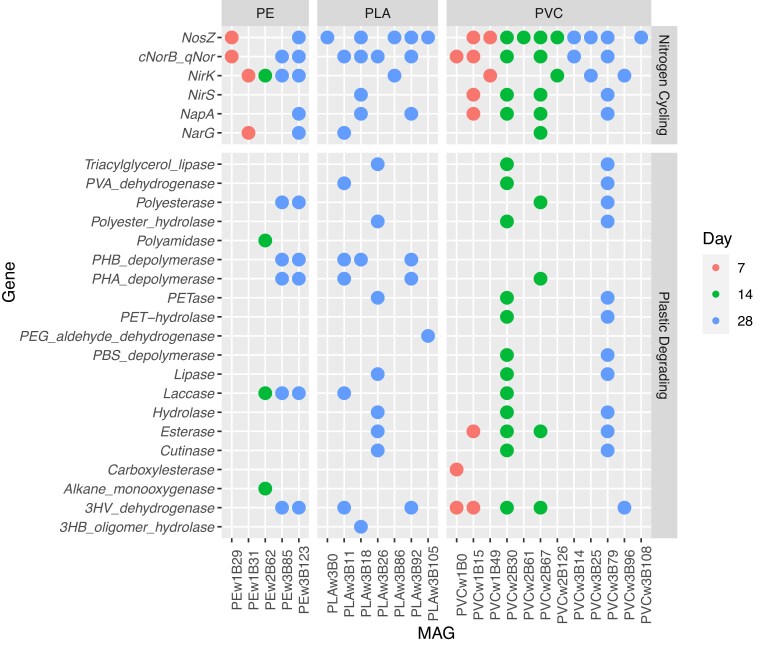
Presence of nitrogen cycling genes and plastic-degrading genes in MAGs. A circle indicates the presence of the gene in the MAG. Color represents the timepoint of the plastisphere each MAG was derived from. The polymers were PE, PLA, and PVC.

Twenty-four MAGs, out of 122 (Supplementary Table S1), contained at least one of the target nitrogen-cycling genes (Table 1). PE had the lowest number of MAGs related to nitrogen metabolism, though at least one MAG was present at every timepoint (Fig. 4). All PLA MAGs were from the final day 28 timepoint. PVC had the highest number of MAGs related to nitrogen metabolism. These were present at every timepoint and increased in number over time (Fig. 4). Across all timepoints and polymers, the MAGs related to nitrogen metabolism were dominated by *Alphaproteobacteria*, including *Rhizobiales* and *Rhodobacterales*, and *Gammaproteobacteria*, including *Burkholdariales* and *Pseudomonadales*. MAGs belonging to *Flavobacteriales* (*Bacteroidia)* were the next most common. Only one MAG, a *Dietzia* species, belonged to the phylum *Actinobacteriota* (Table 1).

**Table 1.** The characteristics of MAGs carrying nitrogen cycling genes, including name (MAG), completeness (%), contamination (%), and taxonomic classification.

Polymer	MAG	Completeness (%)	Contamination (%)	Phylum	Class	Order	Family	Genus
PE	PEw1B29	98.86	1.24	*Bacteroidota*	*Bacteroidia*	*Flavobacteriales*	*Flavobacteriaceae*	*Aequorivita*
	PEw1B31	94.55	3.75	*Proteobacteria*	*Alphaproteobacteria*	*Rhizobiales*	*Rhizobiaceae*	*Hoeflea*
	*PEw2B62	93.78	1.57	*Actinobacteriota*	*Actinobacteria*	*Mycobacteriales*	*Mycobacteriaceae*	*Dietzia*
	*PEw3B85	73.42	2.29	*Proteobacteria*	*Gammaproteobacteria*	*Burkholderiales*	*Burkholderiaceae*	*Pigmentiphaga*
	*PEw3B123	99.53	0.47	*Proteobacteria*	*Gammaproteobacteria*	*Burkholderiales*	*Burkholderiaceae*	*Achromobacter*
PLA	PLAw3B0	93.2	3.73	*Bacteroidota*	*Bacteroidia*	*Flavobacteriales*	*Flavobacteraceae*	GC1-02-35–72
	*PLAw3B11	99.85	2.21	*Proteobacteria*	*Gammaproteobacteria*	*Burkholderiales*	*Burkholderiaceae*	*Massilia*
	*PLAw3B18	87.21	4.62	*Proteobacteria*	*Alphaproteobacteria*	*Rhodobacterales*	*Rhodobacteraceae*	*Paracoccus*
	*PLAw3B26	97.81	1.02	*Proteobacteria*	*Gammaproteobacteria*	*Pseudomonadales*	*Pseudomonadaceae*	*Pseudomonas*
	PLAw3B86	97.85	3.51	*Bacteroidota*	*Bacteroidia*	*Flavobacteriales*	*Flavobacteriaceae*	*Arenibacter*
	*PLAw3B92	98.75	1.98	*Proteobacteria*	*Alphaproteobacteria*	*Rhizobiales*	*Beijerinckiaceae*	*Microvirga*
	*PLAw3B105	100	0.43	*Proteobacteria*	*Alphaproteobacteria*	*Rhizobiales*	*Rhizobiaceae*	*Pseudochrobactrum*
PVC	*PVCw1B0	94.52	4.36	*Proteobacteria*	*Alphaproteobacteria*	*Sphingomonadales*	*Sphingomonadaceae*	*Erythrobacter*
	*PVCw1B15	95.98	0.96	*Proteobacteria*	*Gammaproteobacteria*	*Pseudomonadales*	*Pseudomonadaceae*	*Pseudomonas*
	PVCw1B49	77.56	2.91	*Bacteroidota*	*Bacteroidia*	*Flavobacteriales*	*Flavobacteriaceae*	*Arenibacter*
	*PVCw2B30	100	0.14	*Proteobacteria*	*Gammaproteobacteria*	*Pseudomonadales*	*Pseudomonadaceae*	*Pseudomonas*
	PVCw2B61	88.33	3.72	*Bacteroidota*	*Bactroidia*	*Flavobacteriales*	*Flavobacteriaceae*	CG1-02-35–72
	*PVCw2B67	94.26	0.66	*Proteobacteria*	*Gammaproteobacteria*	*Pseudomonadales*	*Pseudomonadaceae*	*Pseudomonas*
	PVCw2B126	98.48	0.21	*Bacteroidota*	*Bacteroidia*	*Flavobacteriales*	*Flavobacteriaceae*	*Aequorivita*
	PVCw3B14	98.77	1.4	*Bacteroidota*	*Bacteroidia*	*Chitinophagales*	*Chitinophagaceae*	UBA2791
	PVCw3B25	99.09	1.16	*Bacteroidota*	*Bacteroidia*	*Flavobacteriales*	*Flavobacteriaceae*	*Arenibacter*
	*PVCw3B79	98.74	1.5	*Proteobacteria*	*Gammaproteobacteria*	*Pseudomonadales*	*Pseudomonadaceae*	*Pseudomonas*
	*PVCw3B96	95.07	4.74	*Proteobacteria*	*Alphaproteobacteria*	*Rhizobiales*	*Devosiaceae*	*Devosia*
	PVCw3B108	99.57	0.43	*Proteobacteria*	*Alphaproteobacteria*	*Rhizobiales*	*Rhizobiaceae*	*Pseudochrobactrum*

MAG names labeled with a * have plastic-degrading genes.

All MAGs related to nitrogen metabolism obtained from the plastispheres were potential denitrifiers. None of the MAGs contained *nifH, nrfA*, or *amoA* (Fig. 4). In PE, the MAGs were largely incomplete denitrifiers, containing genes for only part of the denitrification process; only one PE MAG, PEw3B123 assigned to *Achromobacter (Burkholderiales)* sp., was a complete denitrifier containing all the genes (i.e. *narG/napA, nirS/K, cnorB_norQ*, and *nosZ*) necessary to convert nitrate to N_2_ gas (Fig. 4). PLA MAGs related to nitrogen metabolism were also mostly incomplete denitrifiers, with only one *napA-*containing complete denitrifier, PLAw3B18. Two of the MAGs contained only *nosZ* (Fig. 4). PVC not only had the highest number of denitrifying MAGs but also the highest number of complete denitrifiers with 4 *Pseudomonas* sp. MAGs containing all the necessary genes (Fig. 4).

**Figure 5.**
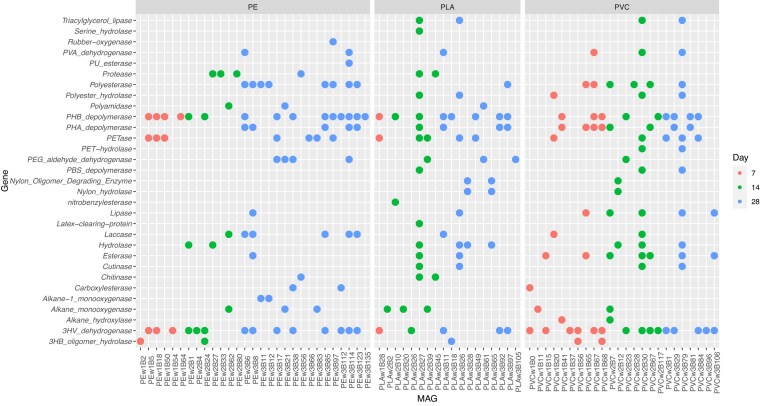
Plastic-degrading genes present in MAGs. A circle indicates the presence of the gene in the MAG. Color represents the timepoint of the plastisphere each MAG was derived from, and the plastic polymers are PE, PLA, and PVC. Some of the data are also represented in Fig. 4.

Seventy-one of the MAGs had plastic-degrading genes (Fig. 5, Supplementary Tables S1, S4, S5, S6). PE had the greatest number of plastic-degrading MAGs followed by PVC then PLA. PLA had the lowest number of MAGs overall (Supplementary Table S1), which could have contributed to the low number of plastic-degrading MAGs. The plastic-degrading MAGs were dominated by *Proteobacteria*, both *Alphaproteobacteria* and *Gammaproteobacteria*, then by *Actinobacteria* and *Bacilli*. The PVC plastisphere was very heavily dominated by *Proteobacteria*, with only 2 *Actinobacteria* and no *Bacilli* plastic-degrading MAGs identified (Supplementary Table S6). PE and PLA both had plastic-degrading MAGs from all three phyla (Supplementary Tables S4 and S5).

There was a wide variety in the identity and number of plastic-degrading genes in each plastic-degrading MAG with 32 different genes found throughout all the plastisphere communities. The mean number of plastic-degrading genes per MAG was three, with individual MAGs having between 1 and 13 different plastic-degrading genes. The most common gene was 3HV dehydrogenase, present in 34 MAGs, closely followed by PHB depolymerase, present in 33 MAGs (Fig. 5, Supplementary Tables S4, S5, S6). PE was the only plastisphere substrate in which MAGs contained alkane-1 monooxygenase, rubber-oxygenase, and PU esterase. Nitrobenzylesterase, serine hydrolase, and latex-clearing protein were only found in MAGs obtained from PLA. Alkane hydroxylase and PET hydrolase were only found in PVC MAGs (Fig. 5).

Fourteen of the MAGs related to nitrogen metabolism contained plastic-degrading genes (Table 1, Figs 4 and 5), including all six complete denitrifiers. Two *Pseudomonas* sp. complete denitrifiers found on PVC contained over ten different plastic-degrading genes (Figs 4 and 5). PE MAGs associated with nitrogen metabolism contained plastic-degrading genes capable of degrading PE, PHA, PHB, nylon, and PBAT (Gambarini et al. 2022) (Figs 4 and 5). This implies that the MAGs associated with nitrogen metabolism could be using PE as carbon sources or that the presence of plastic-degrading genes is an indicator of organisms that are better adapted to survive in plastisphere communities in general. PLA denitrifier MAGs could degrade a few different polymers, including PLA and other biodegradable plastics (Figs 4 and 5). PVC MAGs had the widest range of polymer-degrading genes (Figs 4 and 5), though, at the time of analysis, no gene present in the database has been confirmed to degrade PVC. This may mean that these organisms are not using PVC directly but are instead adapted for the environment present on PVC and other plastic particles, that they are capable of using PVC additives in their metabolism, or that the genes known to degrade other types of plastic may provide an advantage to organisms living on PVC.

## Discussion

The first colonizers of microplastics in the York River were similar regardless of polymer type. PE and PLA plastisphere communities differed from those on PVC on day 14 of the deployment. This was largely driven by the high abundance of taxa in the order *Bacilli* in PLA and PE biofilms. However, by day 28, the more mature communities were all similar in beta diversity. The PVC plastisphere community changed the least over the 28 days of incubation. This suggests either a more stable or a faster development of the “mature” biofilm community. Previous research has shown differences in plastisphere communities between petroleum-based polymers and biopolymers in general, and between PE and PLA, specifically (Dussud et al. 2018, Kirstein et al. 2019, Cheng et al. 2021, Zhang et al. 2021). Thus, the similarity between PLA and PE at all timepoints, and between all three polymers at two timepoints, was unexpected. However, the impact of polymer type on the plastisphere community remains under debate and may be masked by changes driven by season and location (Oberbeckmann et al. 2014, Amaral-Zettler et al. 2015). Our study supports the hypothesis that the biofilm community structure is less impacted by polymer type and more by the environmental characteristics of the ecosystem where the plastic resides, especially as the biofilm matures.

Early colonization was characterized across all plastic types by high abundances of *Bacilli* and *Sphingomonadales*, with lower abundances of *Pseudomonadales* and *Rhodobacterales*. The high relative abundances of *Alphaproteobacteria* and *Gammaproteobacteria* have been observed previously in plastisphere communities (Zettler et al. 2013, Oberbeckmann et al. 2014, Cheng et al. 2021, Zhang et al. 2021); however, *Bacilli* has previously been found in only low concentrations (Kirstein et al. 2019). This difference in dominant community members could be linked to location. Our study was conducted in an estuarine ecosystem, while the majority of plastisphere community research has been performed in open ocean environments (Oberbeckmann et al. 2015, Zhai et al. 2023). *Bacilli* have been found to be associated with estuarine sediments (Selvarajan et al. 2024), and an increase in *Bacillus* has been observed in estuarine sediments exposed to high concentrations of PE (Wang et al. 2024). The elevated abundance of *Bacilli* observed in these plastispheres may be due to more contact with estuarine sediments than previous plastisphere communities have had while developing since, even though the microplastics were suspended in the water column, the York River site where the deployment took place is shallow and turbid. Unlike the prokaryote-dominated plastispheres in our study, previous studies found eukaryotes to be the major plastisphere community members (Bryant et al. 2016, Zhang et al. 2021). Eukaryotes may be later colonizers of microplastic particles and require longer than the 28 days in this study to become dominant biofilm community members.

Chesapeake Bay water column microbial communities are often dominated by *Proteobacteria, Actinobacteria, Cyanobacteria*, and *Planctomycetes* (SAR11) (Wang et al. 2020). Water column community composition samples were not collected during the deployment, so direct comparison of the *in situ* water column microbial community with each plastisphere community is not possible. However, water column community composition samples were collected for a different study (Fortin et al. 2022) during the last week of the deployment (Aug 1) at the same location as the deployment (Out of Bloom Sample C from Fortin et al. 2022). That sample showed the water column community was dominated by *Synechococcales*, SAR11, and *Flavobacteriales*, with smaller portions of *Actinomarinales* and *Opitutales* (Fortin et al. 2022). The difference in water column and plastisphere communities observed in the York River agrees with previous research findings, which found that the plastisphere is distinct from other microbiomes (Zettler et al. 2013, Oberbeckmann et al. 2015, Oberbeckmann et al. 2016). Even though the York River microbiome, both water column and sediment, was the source of microbes for the plastisphere community, the plastisphere community experienced different drivers, resulting in a very different community.

Chesapeake Bay water column communities have been found to be influenced by salinity and temperature, as well as chlorophyll *a* and nutrient availability with the most stable and diverse communities found in the summer (Wang et al. 2020). Since the deployment was only 28 days and there was a fairly constant temperature and salinity, there was likely little influence of either temperature or salinity on the plastisphere community other than determining which microbes were present during initial colonization. The increase in chlorophyll *a* during the last two weeks of the deployment was due to a harmful algal bloom that was beginning to develop around 1 August 2017 (Fortin et al. 2022). The lack of phytoplankton sequences overall and the absence of sequences associated with the harmful algal species present in the York River estuary imply little specific influence of the algal bloom on the plastisphere community at the final timepoint, though changing conditions in nutrients and organic matter could influence the plastisphere community. More research needs to be done to directly link changes in plastisphere communities to specific environmental conditions.

MAGs carrying plastic-degrading genes were found in all timepoints and polymers. These plastic-degrading genes have been linked to the degradation of numerous polymers including PE, PET, and PLA. Previous studies have found hydrocarbon degradation genes in PE and PP-based plastisphere communities, and they have been predicted to be found on PE, PP, and PLA (Bryant et al. 2016, Zhang et al. 2021), though most studies have not examined PVC communities for plastic or hydrocarbon degradation. Plastic-degrading genes have also been found in metagenomes from sediments containing high levels of PE (Wang et al. 2024).

Little is known about plastic-degrading organisms, their genetic makeup, and their taxonomy, though research into these organisms is ongoing. Current research into plastic degraders has found evidence that the genera *Pseudomonas, Bacillus, Cellulosimicrobium, Rhodococcus, Ideonella, Psychrobacter*, and *Tenacibaculum* all contain organisms capable of degrading polymers, including the petroleum-based low-density PE and PET, and the biopolymer poly(ε-caprolactone) (PCL) (Yoshida et al. 2016, Dang et al. 2018, Muhonja et al. 2018, Urbanek et al. 2018). Potential plastic-degrading MAGs included organisms belonging to *Pseudomonas, Cellulosimicrobium, Bacillus, Rhodococcus*, and *Psychrobacter*. This confirmed that known plastic-degrading bacteria were growing in the plastisphere communities in the York River. Additional plastic-degrading MAGs belonging to other genera revealed new potential plastic-degrading bacteria. Since this study was based on metagenomics, the ability of these organisms to degrade the polymeric substrates they were associated with cannot be confirmed. Thus, further research into their ability to actively degrade plastics under different conditions is necessary. It can also not be determined if these organisms can degrade plastic additives leaching from the plastics. However, the presence of plastic-degrading genes in MAGs living on multiple polymers suggests the plastic-degrading community is robust, capable of surviving in estuarine environments, and able to colonize multiple polymer types.

The MAGs from PE, PVC, and PLA show that the plastisphere communities have the potential to contribute to the estuarine nitrogen cycle. Unlike previous reports from PE and PP particles found in the Pacific Ocean (Bryant et al. 2016), few nitrogen fixation genes were present in the York River plastispheres. The York River had nitrate/nitrite and ammonium concentrations above 1 µM for most of August 2017 (Fortin et al. 2022), a higher concentration of fixed nitrogen than was likely present in the Pacific Gyre, potentially contributing to the lack of nitrogen fixation genes in this study. Other research has predicted the presence of nitrification genes in plastispheres on PE, PP, and PLA (Zhang et al. 2021). This agrees with the current study in which nitrification genes were found on all examined plastic polymers. The high prevalence of denitrification genes, and in some cases DNRA, is a novel finding, though changes in denitrification rates and increases in genes associated with denitrification and DNRA have been observed in sediments exposed to microplastics (Seeley et al. 2020, Wang et al. 2024).

The presence of genes for two anaerobic processes, denitrification and DNRA, in the water column provides evidence that the plastisphere biofilm communities, like those on natural particles, create microenvironments. These could decrease oxygen concentrations enough to allow for water column denitrification and DNRA. More research will be needed to determine if these processes are actively performed on the surface of microplastics, since metagenomics can only provide information about the genes present in microbes and not reveal if they are actively in use. Since denitrification genes became more prevalent at later timepoints and the number of MAGs containing denitrification genes increased over time, it likely takes time to build up the micro-anaerobic environments necessary for their survival. This would make denitrification a more common feature of mature biofilm communities. The greater number of denitrifiers present on PVC in the early stages of biofilm formation, and the greater number of complete denitrifiers, may indicate the PVC plastisphere develops faster and creates micro-anaerobic environments more rapidly than those on PE or PLA. The difference in the PVC plastisphere community development, namely its increased stability and the similarity between the initial community and the mature community, supports the hypothesis that polymer type is particularly influential during initial development of the plastisphere community. However, that difference may be later masked by the maturing biofilm.

The development of the plastisphere communities, along with micro-anaerobic environments formed within the biofilm, allows microplastics to serve as habitats for diverse nitrogen-cycling organisms. These microbes may have the genetic capacity to perform both aerobic and anaerobic processes in the aerobic water column, potentially influencing nitrogen cycle dynamics in estuarine surface waters and contributing to the removal of fixed nitrogen by the plastisphere. Notably, most of those nitrogen-cycling organisms contained plastic degradation genes, which may give them a competitive advantage within the plastisphere. The breakdown of plastics or their additives could provide an additional carbon source, supporting denitrification among plastisphere community members. While further research is needed to fully elucidate the role of plastisphere-associated nitrogen-cycling and plastic-degrading microbes in estuarine systems, the plastisphere community must be considered when assessing the broader ecological impacts of microplastic pollution.

## Supplementary Material

fiaf035_Supplemental_File

## Data Availability

Metagenomic sequences and metagenome-assembled genomes are available in NCBI BioProject PRJNA739565.
