# Supplementary material for: Microplastic biofilms as potential hotspots for plastic biodegradation and nitrogen cycling: a metagenomic perspective
Source: FEMS Microbiol Ecol. 2025 Apr 2;101(5):fiaf035. doi: 10.1093/femsec/fiaf035 (PMC11995698; doi:10.1093/femsec/fiaf035)
Supplement: fiaf035_Supplemental_File [file fiaf035_supplemental_file.docx]

**
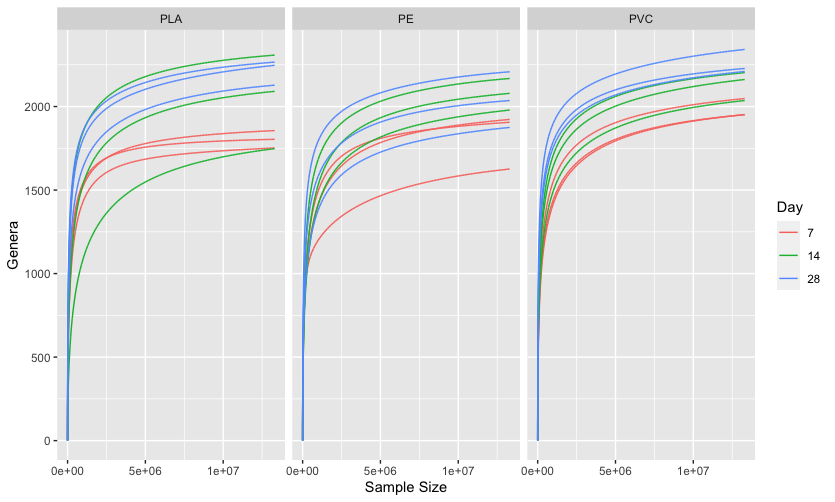
**

**Supplemental Figure 1.** Rarefaction curve showing the number of genera and sample size. Genera identified based on Kaiju identification of sequence reads. PLA: Polylactic acid; PE: Polyethylene; PVC: Polyvinyl chloride.

**Supplemental Table 1.** Table of metagenomic characteristics for each timepoint (Day) and plastic type (Polymer). Read count is the total number of paired reads from the three combined replicates for each day/polymer. Contigs are the number of contigs identified from the assembled reads. High quality metagenome assembled genomes (MAGs) were defined as MAGs with a completeness >70% and <5% contamination.

| *Polymer* | *Day* | *Read Count** | *Contigs* | *High Quality MAGs* | *Nitrogen Cycling MAGs* | *Plastic Degrading MAGs* |
| --- | --- | --- | --- | --- | --- | --- |
| PLA | 7 | 157267808 | 36877 | 4 | 0 | 1 |
| PLA | 14 | 152178628 | 64930 | 13 | 0 | 7 |
| PLA | 28 | 153274822 | 114165 | 17 | 7 | 10 |
| PE | 7 | 158161640 | 73061 | 12 | 2 | 6 |
| PE | 14 | 140337250 | 80276 | 13 | 1 | 7 |
| PE | 28 | 145823398 | 89145 | 22 | 2 | 16 |
| PVC | 7 | 146810186 | 82842 | 14 | 3 | 10 |
| PVC | 14 | 154059166 | 72472 | 15 | 4 | 7 |
| PVC | 28 | 155268612 | 126097 | 12 | 5 | 7 |

^*^Read count from after initial quality check and trimming

**Supplemental Table 2.** Table of target nitrogen cycling genes used to identify nitrogen cycling MAGs and their associated enzymes.

| *Gene* | *Enzyme* |
| --- | --- |
| AmoA | Ammonia monooxygenase |
| NifH_AnfH_VnfH | Nitrogenase reductase |
| NrfA | Cytochrome c-552 nitrite reductase |
| NosZ2 | Nitrous-oxide reductase; Clade 2 |
| NosZ1 | Nitrous-oxide reductase; Clade 1 |
| cNorB_qNor | Nitric-oxide reductase |
| NirK | Nitrite reductase |
| NirS | Cytochrome cd1 nitrite reductase |
| NapA | Periplasmic nitrate reductase |
| NarG | Nitrate reductase |
| Hzo | Hydrazine dehydrogenase |

**Supplemental Table 3.** Environmental conditions of the York River during the microplastic deployment experiment. All values are averages (± standard deviation) of YSI measurements from the Virginia Estuarine and Coastal Observing System continuous monitoring system taken every 15-minutes located in the York River Estuary (Station YRK005.40).

|  | July 11 – Aug 8  *Day 1 – 28* | July 11 – July 18  *Day 1 – 7* | July 19 – 25  *Day 8 – 14* | July 26 – Aug 8  *Day 15 – 28* |
| --- | --- | --- | --- | --- |
| Water Temperature (^o^C) | 27.73 ± 0.94 | 27.89 ± 0.70 | 28.68 ± 0.74 | 27.16 ± 0.68 |
| Salinity (psu) | 20.81 ± 1.21 | 20.62 ± 1.29 | 20.45 ± 1.01 | 21.10 ± 1.19 |
| pH | 7.90 ± 0.21 | 7.79 ± 0.16 | 7.68 ± 0.14 | 7.99 ± 0.22 |
| DO (mg/L) | 6.77 ± 1.89 | 5.87 ± 1.32 | 6.24 ± 1.02 | 7.54 ± 2.16 |
| Turbidity (NTU) | 8.49 ± 15.33 | 7.17 ± 2.95 | 8.52 ± 5.26 | 9.24 ± 21.60 |
| Fluorescence (%FS) | 3.04 ± 15.33 | 1.90 ± 2.95 | 2.18 ± 0.74 | 4.18 ± 7.13 |
| Chlorophyll A (ug/L) | 11.09 ± 18.90 | 6.70 ± 1.72 | 7.63 ± 2.57 | 15.33 ± 26.46 |

**Supplemental Table 4.** Plastic degrading MAGs isolated from polyethylene (PE) plastisphere communities including the identifier (MAG), the MAG quality with the completeness (contamination) scores in percentage, taxonomy, and which plastic degrading genes were present in each MAG.

| **PE Plastic Degrading MAGs** | | | |
| --- | --- | --- | --- |
| *MAG* | *MAG Quality* | *Taxonomy* | *Genes* |
| PEw1B2 | 98.13 (2.75) | Proteobacteria; Alphaproteobacteria; Rhodobacterales; Rhodobacteraceae; Paracoccus | 3HB_oligomer_hydrolase |
| PEw1B5 | 97.84 (2.06) | Proteobacteria; Alphaproteobacteria; Caulobacterales; Caulobacteraceae; Brevundimonas | PHB_depolymerase  3HV_dehydrogenase  PETase |
| PEw1B18 | 81.09 (3.27) | Actinobacteriota; Actinobacteria; Mycobacteriales; Mycobacteriaceae; Rhodococcus | PHB_depolymerase  3HV_dehydrogenase  PETase |
| PEw1B50 | 94.72 (0.27) | Proteobacteria; Alphaproteobacteria; Caulobacterales; Caulobacteraceae; Brevundimonas | PHB_depolymerase  PETase |
| PEw1B54 | 82.52 (3.23) | Proteobacteria; Alphaproteobacteria; Sphingomonadales; Sphingomonadaceae; Sphingorhabdus | 3HV_dehydrogenase |
| PEw1B64 | 92.73 (2.93) | Firmicutes; Bacilli; Bacillales; Bacillaceae | PHB_depolymerase |
| PEw2B1 | 94.74 (4.36) | Proteobacteria; Alphaproteobacteria; Rhizobiales; Anderseniellaceae; Anderseniella | PHB_depolymerase  3HV_dehydrogenase  Hydrolase |
| PEw2B4 | 75.73 (2.77) | Proteobacteria; Alphaproteobacteria; UBA7985; UBA7985 | 3HV_dehydrogenase |
| PEw2B24 | 76.02 (3.97) | Proteobacteria; Alphaproteobacteria; Rhodobacterales; Rhodobacteraceae; Paracoccus | PHB_depolymerase  3HB_oligomer_hydrolase  3HV_dehydrogenase |
| PEw2B27 | 94.4 (2.74) | Firmicutes; Bacilli; Bacillales; Bacillaceae; Bacillus | Protease  Hydrolase |
| PEw2B33 | 90 (1.56) | Firmicutes; Bacilli; Bacillales; Bacillaceae | Protease |
| PEw2B62 | 93.78 (1.57) | Actinobacteriota; Actinobacteria; Mycobacteriales; Mycobacteriaceae; Dietzia | Alkane_monooxygenase  Polyamidase  Laccase |
| PEw2B80 | 97.99 (1.97) | Firmicutes; Bacilli; Bacillales; Bacillaceae; Bacillus | Protease |
| PEw3B6 | 100 (0) | Proteobacteria; Gammaproteobacteria; Burkholderiales; Burkholderiaceae | PHB_depolymerase  3HV_dehydrogenase  Polyesterase  PVA_dehydrogenase  PHA_depolymerase  Laccase |
| PEw3B8 | 97.76 (0.76) | Proteobacteria; Gammaproteobacteria; Pseudomonadales; Pseudomonadaceae; Pseudomonas | 3HV_dehydrogenase  Polyesterase  Esterase  PHA_depolymerase  Laccase  Lipase |
| PEw3B11 | 99.6 (4.87) | Proteobacteria; Gammaproteobacteria; Pseudomonadales; Moraxellaceae; Acinetobacter | Polyesterase  Alkane-1_monooxygenase |
| PEw3B12 | 99.16 (0.14) | Proteobacteria; Gammaproteobacteria; Pseudomonadales; Moraxellaceae; Acinetobacter | Polyesterase  Alkane-1_monooxygenase |
| PEw3B17 | 86.59 (1.56) | Proteobacteria; Alphaproteobacteria; Sphingomonadales; Sphingomonadaceae; Erythrobacter | PHB_depolymerase  PEG_aldehyde_dehydrogenase  3HV_dehydrogenase  PETase |
| PEw3B21 | 96.1 (1.91) | Actinobacteriota; Actinobacteria; Mycobacteriales; Mycobacteriaceae; Rhodococcus | PEG_aldehyde_dehydrogenase  Alkane_monooxygenase  Polyamidase |
| PEw3B38 | 98.13 (2.34) | Proteobacteria; Alphaproteobacteria; Caulobacterales; Caulobacteraceae; Phenylobacterium | PHB_depolymerase  PEG_aldehyde_dehydrogenase  3HV_dehydrogenase  Carboxylesterase |
| PEw3B56 | 78.32 (1.64) | Actinobacteriota; Actinobacteria; Actinomycetales; Cellulomonadaceae; Oerskovia | Chitinase  Protease |
| PEw3B66 | 99.9 (0.55) | Proteobacteria; Gammaproteobacteria; Enterobacterales; Enterobacteriaceae; Leclercia | PETase |
| PEw3B83 | 95.74 (2.11) | Actinobacteriota; Actinobacteria; Mycobacteriales; Mycobacteriaceae; Mycolicibacterium | Alkane_monooxygenase  PETase |
| PEw3B85 | 73.42 (2.29) | Proteobacteria; Gammaproteobacteria; Burkholderiales; Burkholderiaceae; Pigmentiphaga | PHB_depolymerase  3HV_dehydrogenase  Polyesterase  PHA_depolymerase  Laccase |
| PEw3B97 | 79.69 (3.17) | Proteobacteria; Gammaproteobacteria; Pseudomonadales; Cellvibrionaceae; Microbulbifer | PHB_depolymerase  Polyesterase  Rubber-oxygenase  PETase |
| PEw3B112 | 95.8 (1.45) | Proteobacteria; Alphaproteobacteria; Sphingomonadales; Sphingomonadaceae; Sphingomonas | PHB_depolymerase  3HV_dehydrogenase  Carboxylesterase |
| PEw3B114 | 99.12 (3.25) | Proteobacteria; Gammaproteobacteria; Burkholderiales; Burkholderiaceae | PU_esterase  PHB_depolymerase  PEG_aldehyde_dehydrogenase  3HV_dehydrogenase  Polyesterase  PVA_dehydrogenase  PHA_depolymerase  Laccase |
| PEw3B123 | 99.53 (0.47) | Proteobacteria; Gammaproteobacteria; Burkholderiales; Burkholderiaceae; Achromobacter | PHB_depolymerase  3HV_dehydrogenase  Polyesterase  PHA_depolymerase  Laccase |
| PEw3B135 | 97.76 (4.46) | Firmicutes; Bacilli; Bacillales; Bacillaceae | PHB_depolymerase |

**Supplemental Table 5.** Plastic degrading MAGs isolated from polylactic acid (PLA) plastisphere communities including the identifier (MAG), the MAG quality with the completeness (contamination) scores in percentage, taxonomy, and which plastic degrading genes were present in each MAG.

| **PLA Plastic Degrading Genes** | | | |
| --- | --- | --- | --- |
| *MAG* | *MAG Quality* | *Taxonomy* | *Genes* |
| PLAw1B28 | 99.68 (1.1) | Proteobacteria; Alphaproteobacteria; Caulobacterales; Caulobacteraceae; Brevundimonas | PHB_depolymerase  3HV_dehydrogenase  PETase |
| PLAw2B2 | 99.94 (0.5) | Actinobacteriota; Actinobacteria; Mycobacteriales; Mycobacteriaceae; Rhodococcus | Alkane_monooxygenase |
| PLAw2B10 | 93.15 (1.67) | Firmicutes; Bacilli; Bacillales; Bacillaceae; Bacillus | PHB_depolymerase  Nitrobenzylesterase |
| PLAw2B20 | 95.38 (1.28) | Actinobacteriota; Actinobacteria; Mycobacteriales; Mycobacteriaceae; Rhodococcus | Alkane_monooxygenase |
| PLAw2B26 | 99.13 (0) | Proteobacteria; Alphaproteobacteria; Rhizobiales; Rhizobiaceae; Ochrobactrum | 3HV_dehydrogenase |
| PLAw2B27 | 95.67 (1.63) | Actinobacteriota; Actinobacteria; Streptomycetales; Streptomycetaceae; Streptomyces | PHB_depolymerase  PHA_depolymerase  PBS_depolymerase  Esterase  Cutinase  Chitinase  Hydrolase  Serine_hydrolase  Triacylglycerol_lipase  Latex-clearing-protein  PETase  Polyester_hydrolase  Protease |
| PLAw2B39 | 98.57 (3.19) | Actinobacteriota; Actinobacteria; Mycobacteriales; Mycobacteriaceae; Mycolicibacterium | PEG_aldehyde_dehydrogenase  Alkane_monooxygenase  PETase |
| PLAw2B45 | 99.42 (2.7) | Actinobacteriota; Actinobacteria; Actinomycetales; Cellulomonadaceae; Cellulosimicrobium | Chitinase  Protease |
| PLAw3B11 | 99.85 (2.21) | Proteobacteria; Gammaproteobacteria; Burkholderiales; Burkholderiaceae; Massilia | PHB_depolymerase  3HV_dehydrogenase  PVA_dehydrogenase  PHA_depolymerase  Laccase |
| PLAw3B18 | 87.21 (4.62) | Proteobacteria; Alphaproteobacteria; Rhodobacterales; Rhodobacteraceae; Paracoccus | PHB_depolymerase  3HB_oligomer_hydrolase |
| PLAw3B26 | 97.81 (1.02) | Proteobacteria; Gammaproteobacteria; Pseudomonadales; Pseudomonadaceae; Pseudomonas | Polyester_hydrolase  Triacylglycerol_lipase  Cutinase  Lipase  PETase  Esterase  Hydrolase  PETase |
| PLAw3B28 | 97.08 (1.17) | Actinobacteriota; Actinobacteria; Actinomycetales; Microbacteriaceae; Leucobacter | Nylon_Oligomer_Degrading_Enzyme  Nylon_hydrolase  Hydrolase |
| PLAw3B49 | 96.01 (3.61) | Proteobacteria; Alphaproteobacteria; Caulobacterales; Caulobacteraceae; Brevundimonas | PHB_depolymerase  PETase |
| PLAw3B61 | 98.68 (1.13) | Actinobacteriota; Actinobacteria; Mycobacteriales; Mycobacteriaceae; Rhodococcus | PEG_aldehyde_dehydrogenase  Polyamidase |
| PLAw3B65 | 99.49 (3.39) | Actinobacteriota; Actinobacteria; Actinomycetales; Microbacteriaceae; Microbacterium | Nylon_Oligomer_Degrading_Enzyme  Nylon_hydrolase  Hydrolase |
| PLAw3B92 | 98.75 (1.98) | Proteobacteria; Alphaproteobacteria; Rhizobiales; Beijerinckiaceae; Microvirga | PHB_depolymerase  PHA_depolymerase  3HV_dehydrogenase |
| PLAw3B97 | 100 (1.05) | Proteobacteria; Gammaproteobacteria; Xanthomonadales; Xanthomonadaceae; Stenotrophomonas | PHB_depolymerase  3HV_dehydrogenase  Polyesterase  PHA_depolymerase |
| PLAw3B105 | 100 (0.43) | Proteobacteria; Alphaproteobacteria; Rhizobiales; Rhizobiaceae; Pseudochrobactrum | PEG_aldehyde_dehydrogenase |

**Supplemental Table 6.** Plastic degrading MAGs isolated from polyvinylchloride (PVC) plastisphere communities including the identifier (MAG), the MAG quality with the completeness (contamination) scores in percentage, taxonomy, and which plastic degrading genes were present in each MAG.

| **PVC Plastic Degrading MAGs** | | | |
| --- | --- | --- | --- |
| *MAG* | *MAG Quality* | *Taxonomy* | *Genes* |
| PVCw1B0 | 94.52 (4.36) | Proteobacteria; Alphaproteobacteria; Sphingomonadales; Sphingomonadaceae; Erythrobacter | 3HV_dehydrogenase  Carboxylesterase |
| PVCw1B11 | 97.29 (2.02) | Actinobacteriota; Actinobacteria; Mycobacteriales; Mycobacteriaceae; Rhodococcus | Alkane_monooxygenase |
| PVCw1B15 | 95.98 (0.96) | Proteobacteria; Gammaproteobacteria; Pseudomonadales; Pseudomonadaceae; Pseudomonas | 3HV_dehydrogenase  Esterase |
| PVCw1B20 | 98.68 (2.29) | Proteobacteria; Gammaproteobacteria; Pseudomonadales; Moraxellaceae; Psychrobacter | Polyester_hydrolase  Laccase  PETase |
| PVCw1B37 | 82.42 (2.47) | Proteobacteria; Alphaproteobacteria; Sphingomonadales; Sphingomonadaceae; Erythrobacter | 3HV_dehydrogenase |
| PVCw1B41 | 95.73 (3.14) | Proteobacteria; Gammaproteobacteria; Xanthomonadales; Xanthomonadaceae; Lysobacter | PHB_depolymerase  Alkane_hydroxylase  PHA_depolymerase |
| PVCw1B56 | 91.76 (4.43) | Proteobacteria; Alphaproteobacteria; Rhodobacterales; Rhodobacteraceae; Paracoccus | 3HB_oligomer_hydrolase  3HV_dehydrogenase |
| PVCw1B65 | 99.68 (0.81) | Proteobacteria; Gammaproteobacteria; Pseudomonadales; Pseudomonadaceae; Pseudomonas | Polyesterase  Esterase  PHA_depolymerase  Lipase |
| PVCw1B67 | 99.55 (1.36) | Proteobacteria; Gammaproteobacteria; Burkholderiales; Burkholderiaceae; Massilia | PHB_depolymerase  3HV_dehydrogenase  Polyesterase  PVA_dehydrogenase  PHA_depolymerase |
| PVCw1B68 | 80.44 (4.27) | Proteobacteria; Alphaproteobacteria; Rhodobacterales; Rhodobacteraceae; Pseudooceanicola | PHB_depolymerase  3HB_oligomer_hydrolase  3HV_dehydrogenase  PHA_depolymerase |
| PVCw2B7 | 99.84 (0.65) | Proteobacteria; Gammaproteobacteria; Pseudomonadales; Pseudomonadaceae; Pseudomonas | Alkane_hydroxylase  Polyesterase  Alkane_monooxygenase  Esterase  PHA_depolymerase  Lipase |
| PVCw2B12 | 93.33 (4.84) | Actinobacteriota; Actinobacteria; Actinomycetales; Microbacteriaceae; Microbacterium | Nylon_Oligomer_Degrading_Enzyme  Nylon_hydrolase  Hydrolase |
| PVCw2B23 | 84.94 (2.43) | Proteobacteria; Alphaproteobacteria; Rhizobiales; Rhizobiaceae; Rhizobium | PHB_depolymerase  PEG_aldehyde_dehydrogenase  3HV_dehydrogenase |
| PVCw2B28 | 97.41 (0) | Proteobacteria; Gammaproteobacteria; Pseudomonadales; Halomonadaceae; Cobetia | Polyesterase |
| PVCw2B30 | 100 (0.14) | Proteobacteria; Gammaproteobacteria; Pseudomonadales; Pseudomonadaceae; Pseudomonas | PBS_depolymerase  3HV_dehydrogenase  PVA_dehydrogenase  PET-hydrolase  PETase  Polyester_hydrolase  Triacylglycerol_lipase  Cutinase  Lipase  Esterase  Hydrolase  Laccase |
| PVCw2B67 | 94.26 (0.66) | Proteobacteria; Gammaproteobacteria; Pseudomonadales; Pseudomonadaceae; Pseudomonas | 3HV_dehydrogenase  Polyesterase  Esterase  PHA_depolymerase |
| PVCw2B117 | 98.98 (2.08) | Proteobacteria; Alphaproteobacteria; Rhizobiales; Rhizobiaceae; Nitratireductor | PHB_depolymerase  3HV_dehydrogenase |
| PVCw3B1 | 97.4 (3.23) | Proteobacteria; Alphaproteobacteria; Caulobacterales; Caulobacteraceae; Brevundimonas | PHB_depolymerase  3HV_dehydrogenase  PETase |
| PVCw3B29 | 81.04 (2.2) | Proteobacteria; Alphaproteobacteria; Sphingomonadales; Sphingomonadaceae; Sphningosinicella | PHB_depolymerase  3HV_dehydrogenase  PHA_depolymerase |
| PVCw3B79 | 98.74 (1.5) | Proteobacteria; Gammaproteobacteria; Pseudomonadales; Pseudomonadaceae; Pseudomonas | PBS_depolymerase  PETase  Polyesterase  PVA_dehydrogenase  PET-hydrolase  Polyester_hydrolase  Triacylglycerol_lipase  Cutinase  Lipase  Esterase  Hydrolase |
| PVCw3B81 | 100 (0.74) | Proteobacteria; Gammaproteobacteria; Enterobacterales; Alteromonadaceae; Rheinheimera | PHB_depolymerase  PHA_depolymerase |
| PVCw3B84 | 95.07 (4.74) | Proteobacteria; Alphaproteobacteria; Rhizobiales; Devosiaceae; Devosia | PHB_depolymerase  3HV_dehydrogenase  PETase |
| PVCw3B96 | 100 (1.69) | Proteobacteria; Gammaproteobacteria; Pseudomonadales; Pseudomonadaceae; Pseudomonas | 3HV_dehydrogenase |
| PVCw3B106 | 94.52 (4.36) | Proteobacteria; Alphaproteobacteria; Sphingomonadales; Sphingomonadaceae; Erythrobacter | 3HV_dehydrogenase  Esterase  Lipase |
